# Association Between Physical Activity and Suicidal Ideation: The Moderating Effect of Self-Rated Health Status

**DOI:** 10.3390/healthcare13131506

**Published:** 2025-06-24

**Authors:** Da-Jeong Lee, Ki-Soo Park

**Affiliations:** 1 Department of Bio & Medical Big Data, Gyeongsang National University, Jinju 52828, Republic of Korea; leedj@gnu.ac.kr; 2 Department of Preventive Medicine, College of Medicine, Institute of Medical Science, Gyeongsang National University, Jinju 52727, Republic of Korea

**Keywords:** suicide, self-rated health status, physical activity

## Abstract

**Background**: This study aimed to evaluate the association between physical activity and suicidal ideation in adult women and to examine the moderating effect of self-rated health status. **Methods**: This study utilized raw data from the Korea National Health and Nutrition Examination Survey (KNHANES) from the 6th to the 8th cycle (2013–2021). Complex sample logistic regression analysis was performed to assess the association between physical activity and suicidal ideation, and stratified analysis was conducted to examine differences in effects according to self-rated health status. **Results**: The prevalence of suicidal ideation among participants was 5.5%. Stratified analysis revealed that the effect of physical activity on suicidal ideation varied by self-rated health status. Among women with good self-rated health, physical activity was associated with significantly reduced odds of suicidal ideation (OR = 2.116, 95% CI: 1.229–3.643). However, among those with fair or poor self-rated health, physical activity showed no significant protective effect (OR = 1.173, 95% CI: 0.902–1.525). **Conclusions**: This study demonstrates a significant association between physical activity and suicidal ideation, with self-rated health status playing a key moderating role. Suicide prevention strategies should incorporate interventions that promote physical activity while considering self-rated health status.

## 1. Introduction

Suicide is a significant public health concern in South Korea; the suicide rate in South Korea is 25.2 per 100,000 persons [1], with the country having the highest female suicide rate among the OECD countries in 2020 [2]. In 2015, self-harm had the highest socioeconomic burden costs among all diseases and injuries in Korea [3]. The proportion of respondents who experienced suicidal ideation during the past 12 months is 1.3%, with women having a 1.7 times higher prevalence than men [4]. The elevated risk of suicide among women and the high prevalence of suicidal ideation highlight the severity of the issue and emphasize the need for effective prevention and management strategies to address suicide and suicidal ideation in South Korea.

Suicide is a complex phenomenon resulting from the interaction of multiple factors, which can be categorized as non-modifiable and modifiable. Non-modifiable factors include demographic variables, while modifiable factors comprise socioeconomic status, chronic disease, mental health, substance abuse, emotional factors, and interpersonal relationships [5,6,7]. Many of these risk factors for suicide are also known to be risk factors for suicidal ideation. For instance, educational level, income, chronic disease, and depression have been identified as risk factors for suicidal ideation [8,9,10,11]. Therefore, preventing and managing these common risk factors may contribute to the prevention of both suicidal ideation and suicidal behavior.

Physical activity has been shown to have a positive effect on risk factors for suicide, and previous research has demonstrated that physical activity is associated with a reduction in suicidal ideation [12,13]. Previous studies have suggested biological, psychological, and social mechanisms through which physical activity may influence suicidal ideation, including neurotransmitter regulation, hypothalamic–pituitary–adrenal (HPA) axis regulation, self-esteem, social support and integration, and health-related self-efficacy [14,15,16,17,18]. However, the effects of physical activity vary depending on factors such as health status, physical activity preference, and mental illness severity [19,20]. Furthermore, mental illness can be a barrier to engaging in physical activity [21]. It is important to note that the relationship between physical activity and mental health is bidirectional, with each influencing the other. Consequently, understanding the effects of physical activity on mental health requires a consideration of their interaction.

Self-rated health status (SRH) is a robust predictor of various health outcomes. A study conducted on medical patients reported that poor SRH was significantly associated with an increased risk of suicidal ideation, independent of the presence of common mental or physical health conditions [22]. Moreover, SRH demonstrated the strongest association with daily engagement in physical activity [23]. The interaction between SRH and physical activity may influence other health outcomes, emphasizing the need for stratification by SRH when investigating these relationships. Despite the potential implications, previous studies have not focused on the moderating role of SRH in the relationship between physical activity and suicidal ideation. Therefore, the current study aims to examine the role of SRH in the effect of physical activity on suicidal ideation in the general population of women in South Korea.

## 2. Materials and Methods

### 2.1. Study Population

This study used data from the Korea National Health and Nutrition Examination Survey (KNHANES), a nationwide cross-sectional survey conducted by the Korea Centers for Disease Control and Prevention. The survey aims to produce nationally representative statistics on the health and nutritional status of the Korean population.

This study used raw data from the KNHANES phases VI–VIII, covering odd-numbered years from 2013 to 2021. The study population were selected from women aged 19 years or older, excluding those with missing values in the analysis variables. A total of 38,725 individuals participated in the Korea National Health and Nutrition Examination Survey (KNHANES) phases VI–VIII during the odd-numbered years from 2013 to 2021. Among them, 17,349 were women aged 19 years or older. After excluding participants with missing values in all analyzed variables, 12,042 women were included in the study population.

To assess potential selection bias, the sociodemographic characteristics of study population and excluded participants were compared using complex sample crosstabs and chi-square tests. To account for multiple comparisons, Bonferroni correction was applied with an adjusted significance level of *p* < 0.0125. Significant differences were observed in age (*p* < 0.001) (Table 1).

### 2.2. Variables

The following variables were used in the analysis: age, income, education level, cohabitant, monthly alcohol use, current smoking status, physician-diagnosed chronic diseases, and presence of depressed mood for 2 weeks or more.

Age was reclassified into 10-year intervals from 19 to 80 years old. Household income was divided into quintiles (low, low–middle, middle, middle–upper, and upper). Cohabitant status was defined as ‘no’ if the number of household members was 1 and ‘yes’ if it was 2 or more. Monthly alcohol use was defined as having consumed alcohol at least once a month in the past year. Current smoking status (any type of tobacco product) was defined as ‘yes’ for daily or occasional smoking and ‘no’ for former smokers who currently do not smoke or never smokers. The number of chronic diseases was reclassified as none, 1, 2, or 3 or more based on physician-diagnosed hypertension, dyslipidemia, stroke, myocardial infarction, angina pectoris, osteoarthritis, rheumatoid arthritis, gout, pulmonary tuberculosis, asthma, diabetes mellitus, thyroid disease, stomach cancer, liver cancer, colorectal cancer, breast cancer, cervical cancer, lung cancer, thyroid cancer, other cancer 1, other cancer 2, depression, atopic dermatitis, allergic rhinitis, kidney disease, and liver cirrhosis.

SRH was assessed using the question “In general, how do you perceive your health?”. The response options were ‘very good’, ‘good’, ‘fair’, ‘poor’, and ‘very poor’. ‘Very good’ and ‘good’ were defined as ‘good’, while ‘fair’, ‘poor’, and ‘very poor’ were defined as ‘fair’ [24].

Physical activity was defined as engaging in moderate-intensity physical activity for at least 150 min per week, or high-intensity physical activity for at least 75 min per week, or a combination of moderate- and high-intensity physical activity (1 min of high-intensity activity is equivalent to 2 min of moderate-intensity activity) for an equivalent amount of time.

The suicidal ideation in the past year status was assessed using the question “Have you seriously considered suicide in the past year?”. The response options were ‘yes’ and ‘no’.

### 2.3. Statistical Analysis

Due to the complex sampling design of the KNHANES, a complex sample analysis was performed to account for the design elements. Complex samples frequencies were used for general characteristics of participants. Complex samples frequencies were utilized to examine the prevalence of suicidal ideation by SRH and physical activity. Complex samples logistic regression was used to assess the independent association between physical activity, SRH, and suicidal ideation status. All logistic regression models were adjusted for age group, income level, education level, cohabitant status, monthly alcohol use status, current smoking status, number of chronic diseases, and depressed mood using enter method to control for potential confounders. Additional complex samples logistic regression was performed to examine the association of SRH and physical activity with suicidal ideation status. Stratified analysis by SRH was conducted to examine potential effect modification.

All statistical analyses were performed using SPSS 27.0 (IBM Corp., Armonk, NY, USA). A *p*-value < 0.05 was accepted as the significance level.

## 3. Results

### 3.1. General Characteristics

The study sample consisted of 12,042 participants (Table 2). The age distribution was as follows: 17.2% were aged 19–29 years, 17.5% were aged 30–39 years, 19.9% were aged 40–49 years, 19.7% were aged 50–59 years, 13.3% were aged 60–69 years, and 12.4% were aged 70 years and above. The distribution of the number of chronic diseases was as follows: 43.6% had no chronic diseases, 28.3% had one chronic disease, 14.8% had two chronic diseases, and 13.3% had three or more chronic diseases. The depressed mood distribution was as follows: 14.6% reported having a depressed mood and 85.4% reported not having a depressed mood. The SRH distribution was as follows: 71.9% fair and 28.1% good. Regarding physical activity, 37.6% reported engaging in physical activity, while 62.4% did not. The suicidal ideation distribution was as follows: 5.5% reported experiencing suicidal ideation and 94.5% reported no suicidal ideation.

### 3.2. Prevalence of Suicidal Ideation by SRH and Physical Activity

**Figure 1:**
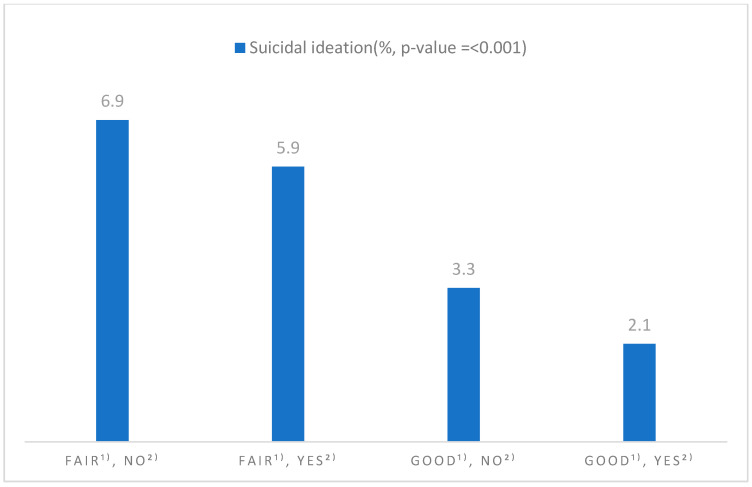
Prevalence of suicidal ideation by self-rated health status and physical activity. (1) Self-rated health status; (2) physical activity.

Suicidal ideation varied significantly across the four groups defined by SRH and physical activity (*p* < 0.001). The prevalence ranged from 6.9% among those with fair SRH who were physically inactive to 2.1% among those with good SRH who were physically active. Intermediate rates occurred in those with fair SRH who were active (5.9%) and those with good SRH who were inactive (3.3%) (Figure 1).

### 3.3. Association Between Physical Activity, SRH, and Suicidal Ideation

In Model 1, physically active individuals had higher odds of being free from suicidal ideation than inactive individuals (OR = 1.301, 95% CI = 1.021–1.657). Model 2 showed that good SRH was associated with reduced suicidal ideation compared to fair SRH (OR = 1.435, 95% CI = 1.063–1.938). Model 3, incorporating both factors, demonstrated that physical activity (OR = 1.283, 95% CI = 1.006–1.637) and good SRH (OR = 1.414, 95% CI = 1.046–1.911) maintained independent associations with absence of suicidal ideation (Table 3).

### 3.4. Association of SRH and Physical Activity with Suicidal Ideation

The combined effects of SRH and physical activity on suicidal ideation were examined (Table 4). Using individuals with good SRH who were physically active as the reference group, all other combinations showed significantly higher risk of suicidal ideation. Those with good SRH who were physically inactive had reduced odds of being free from suicidal thoughts (OR = 0.496, 95% CI = 0.298–0.827), similar to those with fair SRH who were physically active (OR = 0.502, 95% CI = 0.308–0.820). The lowest odds were observed among individuals with fair SRH who were physically inactive (OR = 0.429, 95% CI = 0.270–0.683).

### 3.5. Association Between Physical Activity and Suicidal Ideation Stratified by Self-Rated Health Status

Stratified analysis by SRH revealed differential effects of physical activity (Table 5). Among those with fair SRH, physical activity showed no significant association with suicidal ideation after adjustment for covariates (OR = 1.173, 95% CI = 0.902–1.525). In contrast, among those with good SRH, physically active individuals had more than twice the odds of being free from suicidal ideation compared to inactive individuals (OR = 2.116, 95% CI = 1.229–3.643).

## 4. Discussion

In this nationwide cross-sectional study of 12,042 adult women in South Korea, we examined how SRH modifies the relationship between physical activity and suicidal ideation. Both physical activity and good SRH demonstrated independent associations with reduced suicidal ideation, with physical activity increasing the likelihood of being free from suicidal thoughts by 30.1% and good SRH by 43.5%. However, stratified analysis revealed marked differences in these associations across SRH groups. Among individuals with good SRH, physical activity more than doubled the likelihood of avoiding suicidal thoughts, whereas no such benefit was observed among those with fair SRH. Using combined SRH and physical activity categories, we found the lowest risk of suicidal ideation among those with good SRH who were physically active, while those with fair SRH who were inactive showed the highest risk—representing a more than three-fold difference in prevalence.

The prevalence of suicidal ideation in the study population was 5.5%. In comparison, the World Health Organization (WHO) World Mental Health Surveys, conducted between 2001 and 2007, reported a prevalence of 2.2% in developed countries and 2.4% in developing countries [25]. The KNHANES 2007–2008 found a substantially higher prevalence of 19% [26]. The current study, using data from 2013–2021, found a prevalence of 5.5%, which is still remarkably high compared to other countries. These findings highlight the need for effective interventions and prevention strategies to reduce suicidal ideation in South Korea. These prevalence rates must be understood within the context of gender-specific mental health barriers. Women’s high prevalence of mental disorders [27,28], gender-based violence [29], and conditions of reproductive health are related to mental disorders [30]. Societal expectations regarding gender roles and beauty standards and discrimination lead to mental health problems. Stigma, financial constraints including gender pay gap [31], and underrepresentation in research [32] are obstacles in access to mental health care. These various barriers affect mental health issues and are linked to poor health status and may consequently influence the effectiveness of physical activity.

Physical activity and SRH showed independent protective associations with suicidal ideation. Physical activity was associated with 28.3% higher odds of being free from suicidal ideation, while good SRH was associated with 41.4% higher odds. The comparable magnitude of these associations suggests that both physical activity and health status play important roles in mental health.

The absence of protective effects from physical activity with fair SRH warrants careful consideration of alternative intervention approaches. Supporting this finding, a meta-analysis of adolescents found that physical activity components in interventions showed no overall effect on anxiety, depression, and stress outcomes [33]. Similarly, another meta-analysis examining hospitalized patients found no conclusive evidence that general physical activity promoting interventions improved functional outcomes [34]. These parallel findings across different populations suggest that when health status is compromised, physical activity alone may be insufficient. Therefore, interventions targeting depression and improving SRH are required for this population.

The lack of protective effects of physical activity among individuals with fair SRH may be explained by several theoretical mechanisms. Underlying health conditions may create both physical and psychological barriers to engaging in physical activity, while simultaneously fostering lower intrinsic motivation and learned helplessness [35,36]. Moreover, individuals with poor SRH may not experience the same physiological benefits from exercise [37]. Physical discomfort, fatigue, and negative illness perceptions may interfere with the protective effects of physical activity [38]. Additionally, compromised exercise self-efficacy may attribute any improvements to external factors rather than their own efforts [39]. These biopsychosocial mechanisms collectively suggest that physical activity interventions require modifications for poor SRH to effectively reduce suicidal ideation.

For individuals with fair SRH, physical activity programs may need substantial adaptations to reduce barriers and enhance accessibility. Structural modifications should focus on reducing financial barriers [40], minimizing time and location constraints [41], and offering diverse exercise modalities to accommodate individual preferences [42]. Program design adaptations should include low-difficulty exercises, reduced intensity and duration requirements, incorporation of foundational elements such as stretching, and education regarding the potential benefits of physical activity [43]. Psychological support should emphasize motivation enhancement and reinforcement [44], facilitate online or offline support groups [45], help develop self-regulation skills to improve self-efficacy and perceived control [46], and assist the integration of exercise into daily routines to promote long-term adherence [47]. Such adapted approaches may help bridge the gap between SRH and the potential mental health benefits of physical activity.

In contrast to those with fair SRH, individuals with good SRH showed marked benefits from physical activity engagement. Previous meta-analytic evidence supports this differential response. A meta-analysis of people with mental illness found that physical activity improved depressive symptoms, anthropometric measures, aerobic capacity, and quality of life [48]. Another meta-analysis found a significant negative association between physical activity levels and suicidal ideation [49]. These results suggest that promoting physical activity and implementing interventions to increase physical activity levels are necessary for those with good SRH to reduce the risk of suicidal ideation.

The interaction between SRH and physical activity revealed important patterns. Among those with good SRH, physical inactivity was associated with significantly higher risk of suicidal ideation compared to their physically active counterparts. This highlights that good health perception alone may be insufficient without accompanying health behaviors. Notably, individuals with fair SRH showed similar levels of suicidal ideation regardless of their physical activity status, further confirming that exercise does not confer the same mental health benefits in this group. Previous research aligns with these findings, showing that poor SRH itself is a strong risk factor for suicidal ideation among adolescents [50] and that self-rated mental health status significantly predicts suicidal ideation [51]. These studies reinforce the theory that the mental health benefits of physical activity depend on one’s SRH.

In conclusion, our findings suggest that physical activity has a direct effect on reducing suicidal ideation in individuals with good SRH. However, this effect was not observed in those with fair SRH. This discrepancy may be attributed to the possibility that physical activity influences health status, and through the improvement of health status, it may consequently lead to a reduction in suicidal ideation.

The cross-sectional design of this study limits the ability to make causal inferences. As secondary data were utilized, potential confounders such as hopelessness could not be considered due to the absence of relevant data. The self-reported nature of key variables (physical activity, SRH, suicidal ideation, and depressed mood) may introduce recall and reporting bias. However, the large sample size and representative population provide a basis for suggesting a new focus for effective physical activity interventions that consider individual differences. Longitudinal studies are needed to establish temporal and dose–response relationships between physical activity and suicidal ideation. Objective measures of physical activity, such as accelerometers, should be employed to provide more accurate assessments. Furthermore, intervention studies are required to evaluate the effectiveness of physical activity in reducing suicidal ideation. Future research could explore integrating advanced analytical methods, including machine learning approaches, with traditional epidemiological methods being used to enhance suicide risk prediction.

## 5. Conclusions

Fair SRH and physical activity were not found to have a significant impact on suicidal ideation; however, among those with good SRH, physical activity was found to significantly influence suicidal ideation. For individuals with fair SRH, interventions targeting depression and physical health should be prioritized, while those with good SRH may benefit from additional interventions to promote physical activity. Given the high prevalence rate of suicidal ideation, it is necessary to strengthen physical activity interventions that are effective in suicide prevention. To achieve this, the influence of SRH on the relationship between physical activity and suicide should be considered.

## Figures and Tables

**Table 1:** Sociodemographic characteristics of study population and excluded participants.

	Study Population		Excluded Participants		
	N	Weighted %	N	Weighted %	*p*-Value
Age					<0.001
19–29	1418	17.2	439	15.2	
30–39	1793	17.5	624	15.7	
40–49	2192	19.9	793	18.6	
50–59	2406	19.7	862	18.9	
60–69	2140	13.3	800	13.8	
70≤	2093	12.4	1056	17.7	
Income level					0.217
Low	2326	19.3	960	21.6	
Low–middle	2388	19.7	908	20.0	
Middle	2461	20.2	868	18.8	
Middle–upper	2450	20.6	864	19.6	
Upper	2417	20.1	870	20.0	
Education level					0.365
Elementary school and below	3187	19.6	872	19.7	
Middle school	1226	9.0	327	7.9	
High school	3752	34.2	1061	33.0	
College and above	3877	37.2	1217	39.3	
Cohabitant					0.014
No	1615	10.1	736	12.0	
Yes	10,427	89.9	3830	88.0	

**Table 2:** General characteristics of the participants.

	N	Weighted %
Age		
19–29	1418	17.2
30–39	1793	17.5
40–49	2192	19.9
50–59	2406	19.7
60–69	2140	13.3
70≤	2093	12.4
Income level		
Low	2326	19.3
Low–middle	2388	19.7
Middle	2461	20.2
Middle–upper	2450	20.6
Upper	2417	20.1
Education level		
Elementary school and below	3187	19.6
Middle school	1226	9.0
High school	3752	34.2
College and above	3877	37.2
Cohabitant		
No	1615	10.1
Yes	10,427	89.9
Monthly alcohol use		
Yes	4741	43.0
No	7301	57.0
Current smoking		
Yes	635	6.2
No	11,407	93.8
Chronic disease		
0	4709	43.6
1	3345	28.3
2	1990	14.8
3 and more	1998	13.3
Depressed mood		
Yes	1795	14.6
No	10,247	85.4
Self-rated health status		
Fair	8830	71.9
Good	3212	28.1
Physical activity		
No	7900	62.4
Yes	4142	37.6
Suicidal ideation		
Yes	668	5.5
No	11,374	94.5
Total	12,042	100.0

**Table 3:** Association between physical activity, self-rated health status, and suicidal ideation.

	Model 1		Model 2		Model 3	
	OR	95% CI	OR	95% CI	OR	95% CI
Physical activity						
No	1				1	
Yes	1.301	1.02–1.66			1.283	1.01–1.64
Self-rated health status						
Fair			1		1	
Good			1.435	1.06–1.94	1.414	1.05–1.91

OR, odds ratio; CI, confidence interval. Suicidal ideation (reference category = Yes). Adjusted for age, income, education, cohabitant, alcohol drinking, smoking, chronic disease, and depressed mood.

**Table 4:** Association of self-rated health status and physical activity with suicidal ideation.

	Suicidal Ideation	
	OR	95% CI
Self-rated health status, physical activity		
Good, Yes	1	
Good, No	0.496	0.298–0.827
Fair, Yes	0.502	0.308–0.820
Fair, No	0.429	0.270–0.683

OR, odds ratio; CI, confidence interval. Suicidal ideation (reference category = Yes). Adjusted for age, income, education, cohabitant, alcohol drinking, smoking, chronic disease, and depressed mood.

**Table 5:** Association between physical activity and suicidal ideation stratified by self-rated health status.

Self-Rated Health Status	Fair			Good		
	OR	95% CI	*p*-Value	OR	95% CI	*p*-Value
Physical activity						
No	1			1		
Yes	1.173	0.902–1.525	0.235	2.116	1.229–3.643	0.007

OR, odds ratio; CI, confidence interval. Suicidal ideation (reference category = Yes). Adjusted for age, income, education, cohabitant, alcohol drinking, smoking, chronic disease, and depressed mood.

## Data Availability

Data were obtained from the following website: https://knhanes.kdca.go.kr/knhanes/eng/main.do (accessed on 1 March 2024).

## Abbreviations

The following abbreviations are used in this manuscript:

SRH	self-rated health status
KNHANES	Korea National Health and Nutrition Examination Survey
WHO	World Health Organization
